# Identification of Cisplatin-Regulated Metabolic Pathways in Pluripotent Stem Cells

**DOI:** 10.1371/journal.pone.0076476

**Published:** 2013-10-16

**Authors:** Louise von Stechow, Ainhoa Ruiz-Aracama, Bob van de Water, Ad Peijnenburg, Erik Danen, Arjen Lommen

**Affiliations:** 1 Department of Toxicology, LACDR, Leiden University, Leiden, The Netherlands; 2 RIKILT - Institute of Food Safety, Wageningen, The Netherlands; 3 Netherlands Toxicogenomics Center, Maastricht, The Netherlands; University of Medicine and Dentistry of New Jersey, United States of America

## Abstract

The chemotherapeutic compound, cisplatin causes various kinds of DNA lesions but also triggers other pertubations, such as ER and oxidative stress. We and others have shown that treatment of pluripotent stem cells with cisplatin causes a plethora of transcriptional and post-translational alterations that, to a major extent, point to DNA damage response (DDR) signaling. The orchestrated DDR signaling network is important to arrest the cell cycle and repair the lesions or, in case of damage beyond repair, eliminate affected cells. Failure to properly balance the various aspects of the DDR in stem cells contributes to ageing and cancer. Here, we performed metabolic profiling by mass spectrometry of embryonic stem (ES) cells treated for different time periods with cisplatin. We then integrated metabolomics with transcriptomics analyses and connected cisplatin-regulated metabolites with regulated metabolic enzymes to identify enriched metabolic pathways. These included nucleotide metabolism, urea cycle and arginine and proline metabolism. Silencing of identified proline metabolic and catabolic enzymes indicated that altered proline metabolism serves as an adaptive, rather than a toxic response. A group of enriched metabolic pathways clustered around the metabolite S-adenosylmethionine, which is a hub for methylation and transsulfuration reactions and polyamine metabolism. Enzymes and metabolites with pro- or anti-oxidant functions were also enriched but enhanced levels of reactive oxygen species were not measured in cisplatin-treated ES cells. Lastly, a number of the differentially regulated metabolic enzymes were identified as target genes of the transcription factor p53, pointing to p53-mediated alterations in metabolism in response to genotoxic stress. Altogether, our findings reveal interconnecting metabolic pathways that are responsive to cisplatin and may serve as signaling modules in the DDR in pluripotent stem cells.

## Introduction

Metabolic changes are associated with a number of complex diseases, including cancer, diabetes and neurological disorders. Often, changes in the abundance of small metabolites are linked to changes in the expression or activity of metabolic enzymes or the complete rewiring of metabolic pathways, as seen for cancer cells, which frequently switch their energy production to aerobic glycolysis (known as Warburg effect) and develop a glutamine addiction [1], [2], [3]. Indeed, mutations in a number of metabolic enzymes were recently related to inherited cancer syndromes [3]. This link between metabolism and disease suggests that metabolomics may be used to identify biomarkers suitable for non-invasive methods to determine disease state, treatment and toxic responses [4].

Changes in metabolism may be linked to stress responses, such as genotoxic stress. Irradiation or chemotherapeutic treatment alters the abundance of metabolites, including for example choline-containing compounds, lipids and several amino acids in cancer cell lines [5], [6]. Interestingly, metabolites excreted by cancer-associated stromal cells can modulate chemosensitivity of cancer cells in a paracrine manner [7]. Recently, the NCI60 panel of tumor cells lines was used to correlate treatment response to platinum drugs with baseline metabolic pathways extracted from metabolomics and transcriptomics [8]. However, integrated approaches aimed at unraveling perturbation of metabolic pathways in response to therapy are currently lacking.

The ability of cells to recognize and respond to DNA damage is of vital importance for the maintenance of an intact genome. The DNA crosslinking drug cisplatin is used as common treatment for various solid tumors, e.g. ovarian, non-small cell lung, head and neck, bladder, colorectal and testicular cancer. Despite initial good responses to therapy, patients often develop resistance to cisplatin treatment and toxicity to healthy tissues (including neuro- and renal as well as gastric toxicity) limits the therapeutic window [9]. Next to direct DNA damage, cisplatin also induces non-genotoxic perturbations, such as oxidative stress by shifting the redox balance through binding to nucleophilic molecules; and ER stress, which has been shown to kill enucleated cells [10], [11]. We have unraveled cisplatin-responsive signaling networks in mouse embryonic stem (ES) cells through integration of functional genomics, phosphoproteomics, and transcriptomics [12], [13]. These studies point to a major role for DNA damage response (DDR) signaling in determining the cellular outcome of cisplatin treatment.

Cancer cells typically have disabled crucial DDR signaling routes and often rewire metabolic pathways [3], [14]. We decided to make use of ES cells to study the DDR using systems wide analyses [12], [13]. These cells have an intact, effective DDR and show robust DNA damage-induced apoptosis. At the same time, they have several features that can be extrapolated to cancer cells, such as the lack of G1/S checkpoint after DNA damage, expression of marker genes (e.g. c-Myc), and a high proliferation rate [15], [16]. In this study, we combined metabolomics and transcriptomics analysis of the response to cisplatin in ES cells. The aim was to integrate affected metabolites with regulated metabolic enzymes in order to delineate alterations in metabolic pathways in response to genotoxic stress in pluripotent stem cells.

## Materials and Methods

### Cell Culture and Materials

HM1 mouse ES cells derived from OLA/129 genetic background (provided by Dr. Klaus Willecke, University of Bonn GE) [17] were maintained under feeder free conditions in GMEM medium containing 10% FBS, 5×10^5^ U mouse recombinant leukemia inhibitory factor (LIF; PAA), 25 U/ml penicillin, and 25 µg/ml streptomycin. For metabolomics analysis and microarrays ES cells were used at passage 22. Cells were confirmed to be mycoplasma-free using the Mycosensor kit from Stratagene. The DNA cross-linker cisplatin (cisplatin; *Cis*-PtCl_2_(NH_3_)_2_) was provided by the Pharmacy unit of University Hospital, Leiden NL. Ammonium acetate (NH_4_Ac), sodium chloride (NaCl) and deuterated chloroform (CDCl_3_) were obtained from Merck (Darmstadt, Germany); methanol (MeOH) and acetone from Biosolve (Valkenswaard, The Netherlands). All chemicals and solvents were purchased in the highest purity available. Ultra-pure water was obtained using the PureLab equipment from Rossmark (Ede, The Netherlands).

### Cell Viability, Apoptosis and Cell Cycle Analyses

To monitor cisplatin-induced cell killing, a cell viability assay using ATPlite 1 Step kit (Perkin Elmer) was performed according to the manufacturer’s instructions followed by luminescence measurement using a plate reader. For cell cycle and apoptosis analysis cells were exposed to vehicle (PBS) or cisplatin for 8 h or 24 h. Floating and attached cells were pooled and fixed in 80% ethanol overnight. Cells were stained using PBS EDTA containing 7.5 mM propidium iodine and 40 mg/ml RNAseA and measured by flow cytometry (FACSCanto II; Becton Dickinson). The number of cells in the different cell cycle fractions (and in sub G0/G1 for apoptotic cells), as seen in Fig. S1 was calculated using the BD FACSDiva software.

### Metabolomics – Sample Preparation

5×10^6^ passage 22 ES cells were plated in 75 cm flasks and medium was replaced 24 h later with 5 µM cisplatin or vehicle control (PBS). After 4 h or 8 h incubation, lysis and fractionation were performed as described [18]. Cells were washed four times in the flask with 10 ml of ice cold 0.9% saline (isotonic) to separate the cells from the cell medium; this was followed by one 10 ml wash using 1.2% ammonium acetate (volatile salt) to eliminate possible excess of non-volatile salt in later sample concentration steps. Lysis was acchieved by osmotic shock in 3 ml of ice-cold pure water after which the flask was scraped; this was repeated 3 times (total volume 9 ml; ice-cold heavy dilution to stop enzyme activity). Lysed cell suspensions were then ultrasonicly treated at 4°C to further ensure cell disruption and protein denaturation. 1 ml of 1 M ammonium acetate was then added to ensure good pelleting of membranes during centrifugation (Hereaus, Labofuge 400 R; 4°C for 60 minutes at 3000 rpm). The membrane-free supernatant containing polar and semi-polar metabolites (polar fraction) was frozen at −80°C and then freeze-dried for 2–3 days. The pellet containing the lipid fraction was used as the apolar fraction. As a further precaution to have sterility, protein denaturation and removal of protein before LC-MS analysis, the polar fraction was resuspended in 1 ml of pure methanol, dried under N2 and then redissolved/resuspended again in 1 ml of 50% methanol/50% pure water. This was then centrifuged for 15 minutes at 4500 g at 4°C to remove any pellet. In a final precipitation step in the supernatant remaining gelatin (used as adhesive for ES cells in the flasks) was removed by adding 4 ml of ice-cold acetone for 10 minutes and then repeating the centrifuge step at 13000 g for 15 minutes at 4°C. The supernatant is then dried overnight under a gentle N2-flow at room temperature after which it was stored at −80°C until use. The apolar fraction was resuspended in 1 M NH_4_Ac (these counter-ions ensure better extraction), freeze-dried, extracted with CDCl_3_, and the organic solvent was evaporated under N_2_ flow. The dried extract was stored at −80°C until use. Five independent biological replicates were examined in each experiment. Metabolomics analysis was performed on the apolar fraction, containing the membranes and intracellular lipids (^1^H-NMR), and the polar fraction containing the polar and semi-polar intracellular metabolites (U-HPLC-Orbitrap-MS). This procedure for sample preparation (extraction, washing efficiency, etc) has been optimized and monitored by ^1^H-NMR previously [18].

### Metabolomics – Measurements

Prior to ^1^H-NMR analysis the dried apolar samples were dissolved in 1 ml of CDCl_3._ of which 0.6 ml of was actually used for ^1^H-NMR analysis. The ^1^H-NMR spectra were recorded at 400.13 MHz at 300.0 (±0.02) K on a Bruker Avance 400 narrow bore using a 5.0-mm probe. The spectrometer settings were the same as described previously [18].

The polar samples were analyzed by ultra-high performance liquid chromatography (U-HPLC)-Orbitrap-MS. For this the dried samples were dissolved in 2 ml of H_2_O; formic acid was added to a final concentration of 0.1%. The injection sequence was randomized as described [19]. U-HPLC was performed on a U-HPLC Accela system (Thermo Fisher Scientific, San Jose, CA, USA), with a 150 mm×2.1 mm UPLC BEH-C18 column with 1.7 µm particles (Waters). Chromatographic conditions were as described [18]. The U-HPLC was directly interfaced to a single stage Orbitrap mass spectrometer (Exactive, Thermo Fisher Scientific). Settings of the Orbitrap mass spectrometer are provided (see Material S1). Data were recorded using Xcalibur software version 2.1.0.1139 (Thermo Fisher Scientific). When identification of mass peaks was needed, samples were subjected to LC-nanomate-Orbitrap-MS analysis using chromatography conditions as described above. These identification procedures are as previously described [18]. The raw data obtained in this study are available on request (arjen.lommen@wur.nl).

### Metabolomics - Data Analysis

#### NMR data analysis of apolar samples

A method for normalization of data using phospholipid signals in apolar samples was accessed as previously described [18]. The NMR data were pre-processed and aligned as described using a for Windows updated version of in-house developed software [20]. Based on equal phospholipid content, normalization of data was not required (see Results section). Subsequently, the spreadsheet containing aligned data of the apolar samples was subjected to statistical analysis using Genemaths XT (http://www.applied-maths.com/genemaths/genemaths.htm). Standard initial analysis entailed performing a 2Log transformation and a principal component analysis (PCA) (average of rows and columns subtracted). This was followed by a 2Log transformation, a pre-selection of variables using an ANOVA (p<0.01), followed by a PCA (average of rows and columns subtracted). The grouping in the ANOVA was on the replicates (n = 5) per treatment (control and 5 µM cisplatin) for each timepoint (4 and 8 hours) creating 4 groups with 5 replicates or on the treatments, creating 2 groups of 10 replicates.

#### LC-MS data analysis of polar samples

U-HPLC-Orbitrap-MS data were pre-processed, mass corrected to sub-ppm precision [20] and aligned using MetAlign (http://www.metalign.nl) [21]. In short, this software performs a baseline correction, accurate mass calculation, data smoothing, and noise reduction, followed by alignment between chromatograms. Since the NMR data on apolar samples did not indicate a need for normalization (see Results) and the polar samples were derived from the same cell cultures no normalization was applied on U-HPLC-Orbitrap-MS data. The generated spreadsheet of the dataset (21173 mass peaks) was subjected to statistical analysis using Genemaths XT. Standard initial analysis entailed performing a 2Log transformation and a PCA (average of rows and columns subtracted). This was followed by a 2Log transformation, a pre-selection of variables using an ANOVA (p<0.01; false discovery rate control by the Benjamini & Hochberg procedure), followed by a PCA (average of rows and columns subtracted). The grouping in the ANOVA was: on the replicates for each time point and treatment (4 groups with 5 replicates), per treatment (2 groups of 10 replicates), on the control samples at both time points (2 groups of 5 replicates), as well as per treatment at each time point (2 groups of 5 replicates at 4 h and 2 groups of 5 replicates at 8 h). The peak loadings responsible for the separation in the different PCAs were selected and exported as described [21]. Only those signals with intensity higher than 5 times noise and a fold change higher than 1.2 were taken as candidates for further identification.

#### Identification of metabolites

To facilitate further analysis and identification of the selected signals, GM2MS, an application of MetAlign that re-creates “new chromatograms” only containing the peaks exported from the PCA selection, was used [21]. Polar metabolites were afterwards identified with commercially available standards, using previously acquired identification information [18], with FT-MS/MS analysis (using the LC-nanomate-Orbitrap-MS method described above), and using databases such as the HMDB [http://www.hmdb.ca/] (see Table S1 for the identified metabolites).

### Transcriptomics Analysis and Integration of Metabolomics and Transcriptomics Data

Transcriptional microarray data have been published [12] and are available from ArrayExpress. HM1 ES cells were treated with 10 µM cisplatin or vehicle control for 8 hours in three independent experiments. Total RNA was isolated using the RNAeasy kit (Qiagen) according to manufacturer’s instructions. RNA quality and integrity was assessed with Agilent 2100 Bioanalyzer system (Agilent technologies). Gene expression was measured using Affimetrix MG430 PM Array plates. All raw data passed the affimetrix quality criteria. Normalization of raw data using the robust multi-array average algorithm and statistical analysis was performed using BRBarray tools (http://linus.nci.nih.gov/BRB-ArrayTools.html). The 2269 genes whose expression differed significantly (parametric p<0.0005) between control ES cells and ES cells treated for 8 hours with 10 µM cisplatin were used as input in Ingenuity pathway analysis (IPA) or the cytoscape plug-in Metscape to identify metabolic enzymes [22], [23]. Transcriptional heatmaps were obtained using Multiple Array Viewer (MEV) software. Differentially expressed genes encoding metabolic enzymes and the metabolites that were differentially regulated at 4 h or 8 h (p<0.01) were imported in IPA and Metscape to form integrated metabolic signaling networks and from those retrieve enriched metabolic pathways, based on the criteria “at least three affected molecules, including at least one affected metabolite”.

### RNAi Experiments

siRNA Smartpools were purchased from ThermoFisher Scientific. Cells were transfected with 50 nM Smartpool in 96 well plates using Dharmafect1 transfection reagent (ThermoFisher Scientific). The medium was refreshed after 24 h and cells were exposed to indicated compounds or vehicle controls 48 h post-transfection for 24 h. As readout, a cell viability assay using ATPlite 1 Step kit (Perkin Elmer) was performed according to the manufacturer’s instructions.

### ROS Formation Assay

For probing intracellular ROS, cells were cultured in µClear 96 well plates to 70% confluence. Cells were washed twice with PBS and incubated for 1 h with 40 µM 5-(and-6)-Carboxy-2′,7′-Dichlorofluorescein Diacetate (DCF-DA, Invitrogen) in phenol-red free culture medium. After washing with PBS, cells were exposed to 5 μΜ cisplatin or 250 µM Hydrogen peroxide (H_2_O_2_) in the presence or absence of 10 mM of the ROS scavenger N-acetylcysteine (NAC). Fluorescence was measured at different time points after exposure using a plate reader.

## Results

### Cisplatin-induced Changes on the Metabolome of Embryonic Stem Cells

*General considerations -* To explore intracellular metabolic changes in response to genotoxic stress in pluripotent stem cells, ES cells were treated with a sub-lethal dose of 5 µM cisplatin for 4 h and 8 h and lysates were prepared for metabolomics analysis (Fig. S1A). At 8 h cells began to accumulate in the S/G2 phase of the cell cycle but viability was not significantly affected at these early timepoints while analysis of parallel control plates at later time points confirmed induction of apoptosis by this concentration of cisplatin (Fig. S1B–D). Apolar and polar fractions were collected and used for ^1^H-NMR and U-HPLC-Orbitrap-MS analysis, respectively. To assess whether data normalization was required to correct for potential differences in cell numbers ^1^H-NMR data on the apolar fraction of control and cisplatin-treated ES cells at both time points were analyzed. Since no significant differences (ANOVA p<0.01) in phospholipid content or other apolar metabolites were detected (examples of overlaid spectra are given in Fig. S2), normalization of metabolite data to correct for cell numbers was not required. Fig. S3 shows for the UHPLC-Orbitrap-MS data the PCA after ANOVA (p<0.01; false discovery rate control by the Benjamini & Hochberg procedure); a small subset of 293 mass peaks survive out of a potential of 21173 mass peaks and can be observed to separate the 4 groups in a reproducible way.

**Figure 1:**
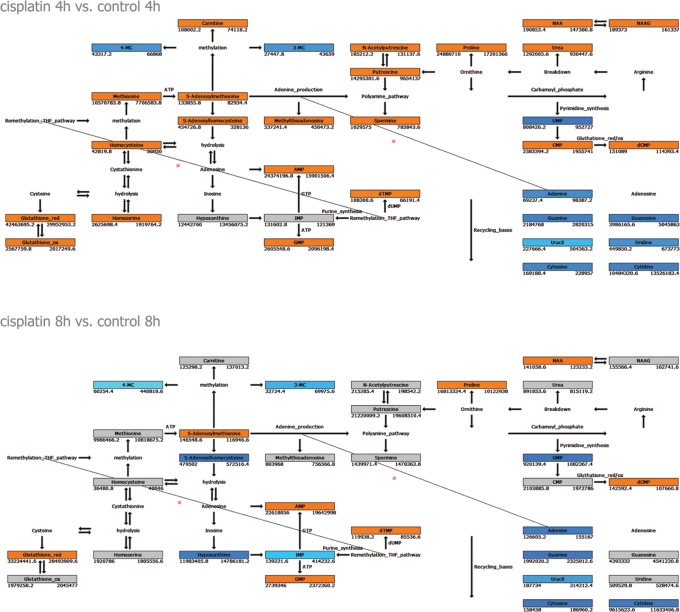
Network of differentially regulated metabolites. Orange, Blue and Grey boxes indicate metabolites (mean of 5 replicates) upregulated, downregulated, and unchanged in cisplatin treatment, respectively (ANOVA p<0.01 and fold change 1.1); metabolites without a box were not detected. Numbers left (cisplatin) and right (control) under boxes indicate the corresponding intensities in MS-data. Results after 4 hours upper panel) and 8 hours (lower panel) are shown.

*Identification of metabolites significantly affected by cisplatin* - Polar fractions of control- and cisplatin-treated samples were compared for each time point independently. Masses were identified that significantly contributed to the observed separation between treatments at the different time points (Table S1). At 4 h, metabolites that were differentially regulated between control and cisplatin-treated samples were mainly involved in methionine degradation pathways (including transmethylation, transsulfuration/glutathione synthesis), as well as polyamine synthesis and catabolism, urea cycle, proline and arginine metabolism, and nucleotide metabolism (Fig. 1A). Furthermore, we detected increased levels of the metabolite N-acetyl-aspartyl-glutamic acid (NAAG), a common neuropeptide and its precursor N-acetyl-L-aspartate (NAA) [24] (Fig. 1A). After 8 h of cisplatin treatment, levels of reduced glutathione and proline remained increased, while other differentially regulated metabolites were mainly involved in nucleotide metabolism (Fig. 1 B, Table S1).

### Expression of Metabolic Enzymes Significantly Affected by Cisplatin

**Figure 2:**
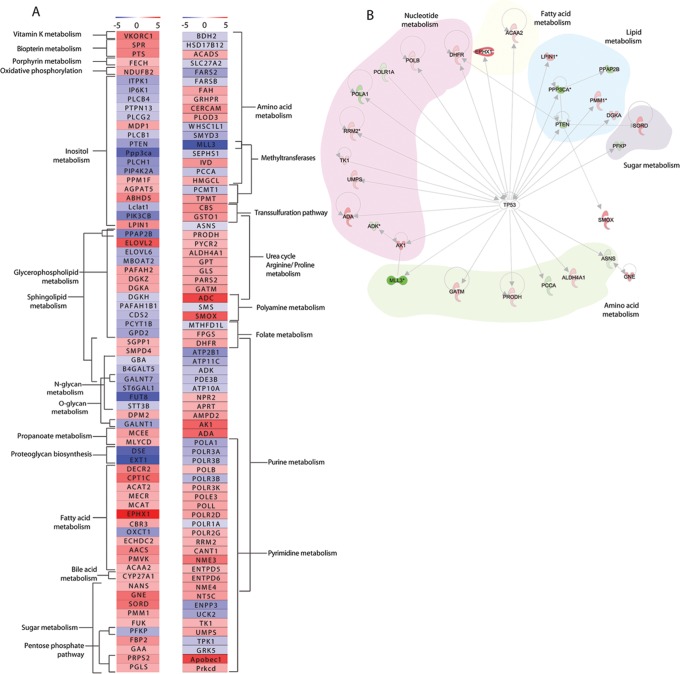
Differentially regulated metabolic enzymes. (**A**) Heatmap indicating metabolic enzymes obtained from Cytoscape metabolic signaling network (Fig. S4, highlighted in blue; Table S2), differentially regulated after 8 h of cisplatin treatment and enriched metabolic pathways within the dataset. (**B**) Regulation of metabolic enzymes by the transcription factor p53 obtained with Ingenuity pathway analysis.

In parallel to metabolomics, transcriptomics analysis was performed to determine cisplatin-induced changes in metabolic enzymes at 8 h. Cytoscape and IPA-based pathway analysis led to the identification of a list of 144 metabolism-related enzymes (Table S2, Fig. 2A). A large proportion of these metabolic enzymes were involved in lipid metabolism; inositol phosphate metabolism (mostly *myo*-Inositol), glycerophospholipid and sphingolipid metabolism. We furthermore detected changes in the mRNA levels of metabolic enzymes that are involved in sugar and fatty acid metabolism (Fig. 2A). Next to those, a number of differentially regulated metabolic enzymes correlated with the metabolic pathways, which had been identified based on the changes in metabolite levels, including urea cycle and arginine/proline metabolism, polyamine metabolism and nucleotide metabolism (Fig. 2A). Interestingly, several of the cisplatin-regulated metabolic enzymes were identified as target genes of the transcription factor p53 (Fig. 2B). p53 target genes implicated in lipid metabolism were commonly suppressed while p53 target genes encoding enzymes functioning in amino acid or nucleotide metabolism were mostly enhanced.

### Identification of Affected Metabolic Pathways through Integration of Metabolomics and Transcriptomics

**Figure 3:**
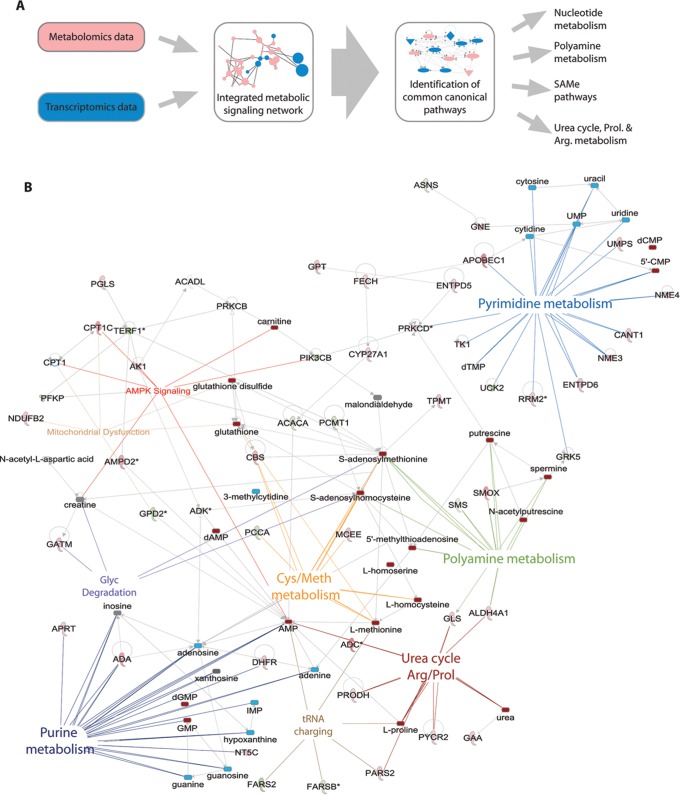
Integrated signaling network. (**A**) Schematic representation of metabolomics and transcriptomics data integration leading to identification of common signaling networks related to nucleotide metabolism, SAMe pathways, polyamine pathways and urea cycle, and arginine & proline metabolism. (**B**) Integrated signaling network of metabolic enzymes and metabolites obtained with Ingenuity pathway analysis. Clusters of significantly enriched canonical pathways and related enzymes and metabolites are highlighted. Upregulated enzymes in green, downregulated enzymes in red. Upregulated metabolites in dark red, downregulated metabolites in blue.

**Figure 4:**
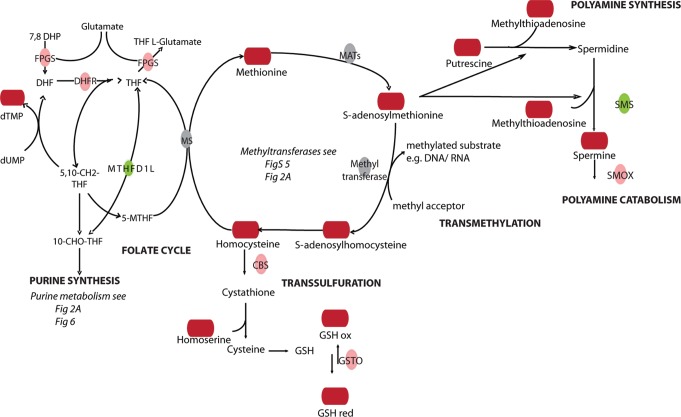
SAMe centered pathways. Upregulated enzymes in green, downregulated enzymes in red. Upregulated metabolites in dark red, downregulated metabolites in blue.

**Figure 5:**
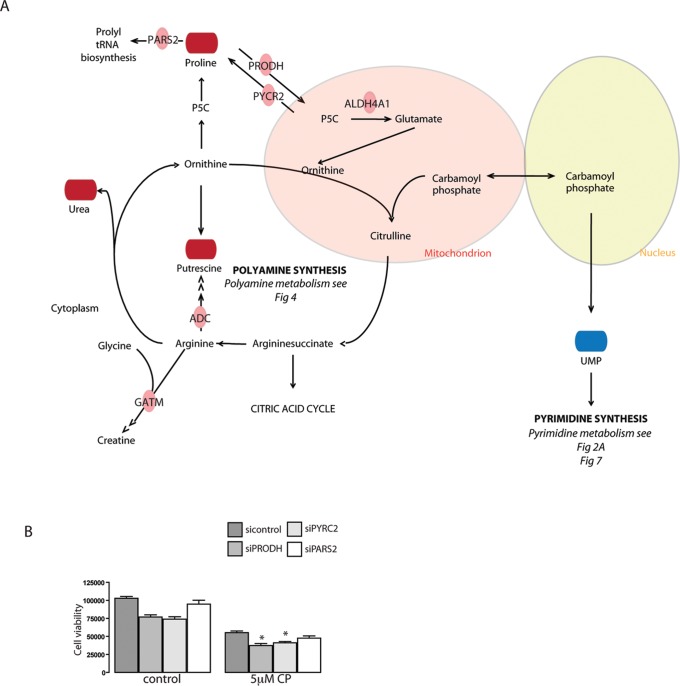
Urea cycle and the metabolism of arginine and proline. (**A**) Upregulated enzymes in green, downregulated enzymes in red. Upregulated metabolites in dark red, downregulated metabolites in blue. (**B**) Cell viability after knockdown of GFP (negative control), PRODH, PYCR2 or PARS2 in presence or absence of 5 µM cisplatin.

**Figure 6:**
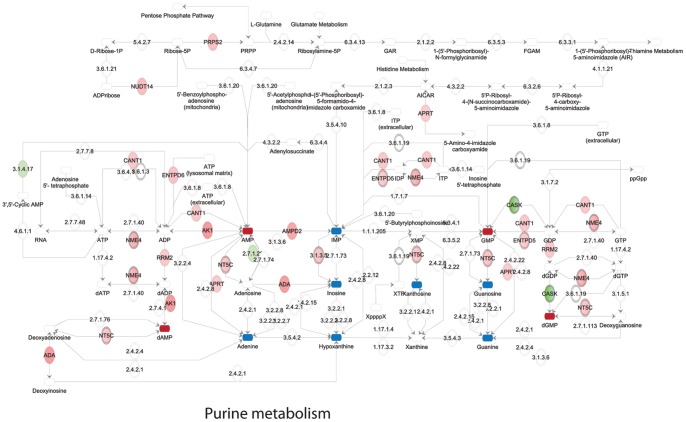
Purine metabolism. Upregulated enzymes in green, downregulated enzymes in red. Upregulated metabolites in dark red, downregulated metabolites in blue.

**Figure 7:**
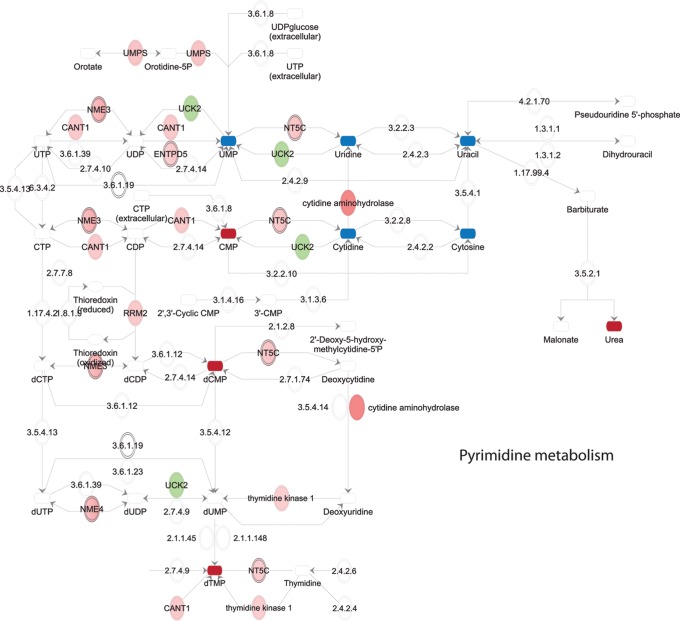
Pyrimidine metabolism. Upregulated enzymes in green, downregulated enzymes in red. Upregulated metabolites in dark red, downregulated metabolites in blue.

Identified changes in metabolites and metabolic enzymes were combined to derive integrated signaling networks. For this, 144 regulated enzymes and 35 regulated metabolites were imported in IPA and Metscape to form an integrated metabolic signaling network (Fig. 3; Fig. S4). Clusters of significantly enriched metabolic pathways were identified based on the criteria [>3 affected molecules including at least 1 affected metabolite and 1 affected enzyme]. Lipid metabolism, despite the observed changes in expression of several enzymes in this process (Fig. 2A; Table S2), was not selected since the sample analysis methodology (NMR) used here for the apolar (lipid) fraction does not have the required resolution for detection of lipd metabolites on the individual species level (such as for instance PIP3 etc). Networks included a purine and a pyrimidine metabolism cluster, a cluster of S-adenosylmethionine (SAMe)-related pathways, a polyamine synthesis cluster, and a urea cycle cluster, featuring pathways related to the metabolism of proline, arginine and citrulline (Fig. 3, 4, 5, Fig. 6, 7).

At 4 h of cisplatin treatment many metabolites linked to SAMe-related pathways were increased (Fig. 1,3,4). SAMe is the methyl donor in methylation of DNA, RNA and methylation of lysine during biosynthesis of carnitine [25]. We detected cisplatin-induced changes in the levels of the RNA nucleosides 3-methylcytidine, 4-methylcytidine, and of carnitine, as well as changes in mRNA levels of the methyltransferases MLL3, PCMT1 and TPMT (Fig. 2A, Fig. 3). We reinvestigated the list of differentially regulated genes and identified regulation of several other methylation enzymes that had been missed by IPA and Metscape analysis, including histone methyltransferases and demethlyases as well as RNA methyltransferases (Fig. S5A). This additional list included the RNA methyltransferase METLL6, which has been correlated to cisplatin sensitivity in lung cancer patients [26]. SAMe is a critical hub between trans-sulfuration, polyamine synthesis, and the folate cycle [25]. Multiple enzymes and metabolites in these pathways were significantly regulated by cisplatin (Fig. 4). This included an increase in cystathionine-beta-synthase (CBS), an enzyme that is critical for the conversion of homocysteine to cystathione [25]. Cystathione is a precursor for glutathione and levels of oxidized and reduced glutathione were increased after 4 h of cisplatin treatment, while after 8 h only the reduced form persisted (Fig. 1, Table S1). Enzymes related to tetrahydrofolate (THF) synthesis, which is a crucial part of the folate cycle were also affected. THF is not only involved in transmethylation, but is also crucial to pyrimidine and purine synthesis. Amongst the regulated enzymes we found upregulation of FPGS and DHFR, which are directly involved in THF synthesis, and downregulation of MTHFD1L, which is involved in the ATP/ADP-dependent interconversion of 10-formyl-THF (needed for purine synthesis) (Fig. 4).

A cluster of reactions related to polyamine synthesis and catabolism were identified based on cisplatin-regulation of enzymes and metabolites (Fig. 4,5). After 4 h of cisplatin treatment we identified increased levels of the polyamine metabolites spermine and its precursor putrescine. At 8 h the concentrations were normalized again, while at the same time spermine synthase (SMS) mRNA levels were reduced and spermine oxidase (SMOX) mRNA levels were increased. In addition, changes in metabolites and enzymes related to urea cycle and proline/arginine metabolism were identified (Fig. 1,5). Arginine catabolism appeared to be enhanced even though we did not observe transcriptional changes in superoxide dismutase or NO-synthase. ADC was found to be strongly increased, potentially resulting in a higher conversion of arginine to agmatine, which itself is an inhibitor of NO-synthase. Moreover, expression of GATM, which forms a creatine precursor from arginine and glycine was enhanced (Fig. 5) [27]. Enhanced levels of metabolic products of arginine catabolism such as urea, putrescine (and spermine) and proline were also detected [27] (Fig. 5). Next to increased proline levels at 4 h and 8 h of treatment, enzymes involved in reduction of P5C to proline (PYCR2), as well as proline breakdown (PRODH, Aldh4a1) were upregulated [28]. IPA analysis identified PRODH, Aldh4a1, and GATM as known p53 target genes [29], [30] (Fig. 2A). Interestingly, altered proline metabolism appeared to act as an adaptive response since knockdown of PYCR2 or PRODH, significantly sensitized ES cells to cisplatin-induced cell death (Fig. 5B).

Since many of the enzymes and metabolites have been previously implicated in anti- or prooxidant function (e.g. glutathione, methionine, proline, PRODH, SMOX) we tested whether cisplatin treatment, at the time points of our analysis, led to an increase in reactive oxygen species (ROS) formation. However, while hydrogen peroxide (H_2_O_2)_ led to a strong increase in ROS levels, no significant increase in intracellular ROS levels was detected after cisplatin treatment, at any of the studied time points (Fig. S5B).

Lastly, several metabolites and metabolic enzymes involved in purine/pyrimidine metabolism were significantly regulated by cisplatin treatment (Fig. 1,2,3,6,7). These were implicated in de novo synthesis and salvage pathways (Fig. 6,7) and included a group of enzymes encoded by genes that were identified in IPA as p53 targets. For instance, DNA polymerase β and RRM2 were upregulated while the catalytic subunit of the replicative B-family DNA polymerase α was downregulated [31], [32] (Fig. 2B).

## Discussion

The DDR activates, in a damage-specific fashion, the appropriate enzyme complexes dedicated to the repair of a variety of DNA lesions. It is critical that the DDR is integrated with ongoing cellular activities, including cell cycle progression, transcription, and translation. In the case of severe damage it is important to arrest the cell cycle, prevent aberrant transcription and translation, preserve energy, and, if damage is beyond repair, to activate cell death mechanisms. This is particularly relevant in stem cells where defective DDR signaling can have major impacts on ageing and cancer [33], [34]. Clearly, the DDR extends into all vital cellular processes. Our current study identifies alterations in metabolic pathways that are likewise controlled by the DDR in pluripotent stem cells.

It must be noted that the metabolomics profiles generated here provide a snapshot of the total dynamic regulation of metabolism under standard and stressed conditions This is for instance due to technical limitations that exclude certain metabolites from the analysis, the labile nature of some metabolites, and the absence of information on fluxes. Nevertheless, the strong reproducibility of the metabolomics and the connections with affected metabolic enzymes provide high-confidence data on the metabolic response of ES cells to genotoxic insult. Interestingly, a number of metabolic enzymes we find to be transcriptionally modified by cisplatin are target genes of the key DDR transcriptional regulator, p53. This is in agreement with our recent finding that p53 is a major DDR signaling hub in ES cells [12]. Moreover, the integration of transcriptionally regulated metabolic enzymes and significantly affected metabolites allows identification of integrated metabolic networks that are responsive to genotoxic stress. These cluster in pathways centered on purine/pyrimidine metabolism, SAMe-related pathways, polyamine synthesis, and the urea cycle.

In a previous study we identified a p53-independent increase in Wnt signaling as a prosurvival response in ES cells, treated with cisplatin [12]. Interestingly, Wnt signaling has been shown to regulate a number of metabolic processes, including glucose, fatty acid, amino acid and nucleotide metabolism. Wnt-mediated metabolic changes are important for a switch from “quiescent” to a “proliferating” metabolic state, which is important both for developmental processes and the pathology of cancer [35]. Thus, Wnt signaling, which acts as a pro-survival response, may regulate some of the DNA damage-induced metabolic changes in pluripotent stem cells. This possibile mode of cross talk warrants further study.

### Changes in Methylation Pathways

Our analysis shows an increase in SAMe-related metabolites in response to cisplatin treatment. SAMe is formed from methionine and ATP with the help of methionine adenosyltransferases (MATs) and functions as a general methyl donor in almost all cellular methylation reactions [25]. Methylation patterns (especially those of DNA) change during embryonic development and neoplastic transformation and can be affected by genotoxic stress [36]. RNA-methylation has been associated with structural features, RNA stability and function (e.g. tRNA codon specificity) [37]. Mammalian cells lack the necessary kinases to phosphorylate modified nucleosides into nucleoside triphosphate, which prevents their recycling and incorporation in mRNA [38]. Instead, modified nucleosides are excreted. Enhanced urinary levels of modified RNA nucleosides, including 3-methylcytidine have been suggested as a potential biomarker for certain cancers [39], [40]. We detect changes in the expression of RNA- but not DNA-methyl transferases and decreased levels of methylated RNA nucleosides in response to cisplatin treatment, including 3- and 4-methylcytidine.

### Changes in Transsulfuration Pathways and Folate Cycle

After methyl transfer SAMe is converted into S-adenosylhomocysteine (SAH), which in turn is hydrolyzed to form adenosine and homocysteine. Homocysteine, can be either reduced to cysteine, which functions as a crucial precursor for glutathione (Transsulfuration pathway), or be remethylated to methionine (Folate cycle) [25]. Our integrated metabolic network associated with SAMe points to changes in these pathways. Glutathione - a cytoprotective compound - has been reported to chelate cisplatin and to play a role in copper transporter-mediated cisplatin efflux. Furthermore, glutathione has a function in cellular redox regulation and can act as a protective agent against cisplatin-induced oxidative stress [41], [42], [43]. In accordance with this, GSTO1, a member of the highly conserved omega class of glutathione transferases with dehydroascorbate reductase activity, which has been implicated in resistance to various genotoxic drugs and irradiation, shows increased levels after cisplatin treatment [44], [45]. In addition, cisplatin treatment causes changes in the levels of enzymes associated with folate synthesis and the folate cycle although altered levels of relevant metabolites are not seen. Expression of DHFR and FGPS is upregulated whereas MTHFD1L is decreased, which could provide a connection to the observed changes in purine metabolism through ATP/ADP-dependent interconversion of 10-formyl-THF [46]. Interestingly, antifolates, such as the DHFR interactor methotrexate are used as antineoplastic agents [47].

### Changes in Polyamine Related Pathways

SAMe serves as a precursor for elongation of putrescine to spermidine and from there to spermine [25]. Both putrescine and spermine levels are enhanced in cisplatin-treated cells at 4 h. The increase of putrescine, which can be derived from ornithine, could also be explained by enhanced expression of ADC, an arginine decarboxylase that converts arginine to agmatine, a precursor of putrescine. Spermine is the substrate for SMOX, an enzyme that is upregulated at 8 h by cisplatin and catalyzes the breakdown of spermine to spermidine, 3-aminopropanal, and H_2_O_2_. Platinum drugs have already been shown to regulate enzymes involved in polyamine catabolism, including spermine N1-acetyltransferase (which is not seen in our study) and SMOX [48], [49]. Moreover, SMOX-produced H_2_O_2_ is considered a major source of oxidative stress after induction of polyamine catabolism [49]. Therefore it appears that the initially increased levels of polyamines could later be levelled out by oxidation potentially leading to an increased production of H_2_O_2_.

### Changes in Proline/Arginine Metabolism and Urea Cycle

We show that cisplatin induces substantial changes in metabolites and enzymes related to urea cycle and proline/arginine metabolism including an apparent increase in arginine catabolism. Arginine, proline, glutamate and ornithine are all interconvertable provided that glutamine is available as a precursor of carbamoyl phosphate. This metabolism is the basis for synthesis of nitrogen-containing compounds such as ureum, putrescine, agmatine, creatine and even nitrogen oxide and is furthermore crucial for pyrimidine synthesis. Interestingly, changes in pyrimidine metabolites are seen in response to cisplatin treatment, including an decrease in the pyrimidine synthesis precursor, UMP [27], [50]. The breakdown of proline by PRODH provides electrons, which can be used for ATP production, but also for ROS formation contributing to apoptosis via intrinsic mitochondrial pathways or the extrinsic death receptor pathway [28].Silencing of the enzymes implicated in this network enhanced cisplatin sensitivity, suggesting that increased proline metabolism is an adaptive rather than a toxic response in ES cells.

### Effects of Cisplatin on Purine/Pyrimidine/Nucleotide Metabolism

Cisplatin affects DNA replication and transcription by causing inter- and intrastrand crosslinks and it can bind free nucleotides. Our integrated networks indicate that pathways potentially involved in DNA metabolism, such as purine/pyrimidine and nucleoside/nucleotide metabolism are significantly increased, including both de novo synthesis and salvage pathways. Notably, interpretation of changes in nucleotide metabolism is complicated by the fact that nucleotides, next to serving as building blocks of DNA and RNA, are involved in a great number of cellular signaling functions, as well as energy metabolism [51]. This cluster may be affected by DNA damage in a p53-dependent manner: affected polymerases and enzymes involved in DNA synthesis and repair are regulated by p53.

Notably, although Cavill et al., analyzing the NCI60 panel of cancer cell lines did not analyze metabolic changes in response to treatment, they did uncover a strong correlation between platinum sensitivity and baseline levels of gene transcripts and metabolites involved in nucleotide metabolism pathways, particularly an increase in nucleotide synthesis [8].

### Possible Cisplatin ROS-related Metabolism

Different studies have reported cisplatin-induced oxidative stress but the relative contribution of this to cisplatin cytotoxicity is unclear. Cisplatin can cause oxidative stress by depleting cellular antioxidant defenses due to its binding to nucleophilic molecules such as glutathione, methionine or cysteine-rich proteins [11]. Furthermore, cisplatin-induced (DDR) signaling processes might cause secondary oxidative stress. The cisplatin-regulated metabolic pathways discussed above provide a number of links to generation of oxidative stress. For instance, a generally increased arginine catabolism may lead to nitrogen oxide formation through NO-synthase, which has been associated with ROS, while an increase in SMOX can lead to H_2_O_2_ production [49]. Notably, we do not detect elevated ROS at the timepoints and cisplatin concentrations used in our study (whereas H_2_O_2_ strongly induces ROS, as expected). This may indicate absence of cisplatin-induced ROS production or effective scavenging of ROS in ES cells. The enhanced levels of glutathione, methionine and proline metabolites may point to the latter explanation and indicate that ES cells cope with cisplatin-induced oxidative stress [43], [52]. However, the fact that the ratio reduced versus oxidized glutathione is slightly elevated in cisplatin-treated cells, argues against glutathione-mediated ROS-scavenging.

### Conclusions

By integrating metabolomics and transcriptomics analyses of cisplatin-treated ES cells, we have identified metabolic pathways that are significantly affected by the treatment with this genotoxic compound. Part of this metabolic response is mediated by p53, in agreement with its central role in the DDR. This includes for instance DNA damage repair-related nucleotide metabolism enzymes (e.g. RRM2) and amino acid catabolic enzymes (e.g. PRODH, ALDH4a1, GATM). Changes in individual metabolic enzymes or metabolites have been reported by others to be responsive to genotoxic stress and/or associated with sensitivity to genotoxic therapy in cancer cells. This holds true for instance for GSTO1, METLL6, PRODH, and SMOX enzymes and metabolites such as carnitine, methionine, and glutathione. Our current study for the first time integrates these events into cisplatin-regulated metabolic signaling networks.

## Supporting Information

Figure S1 **Cisplatin does not lead to cell death at 4 h and 8 h of treatment, but causes cell cycle arrest. (A)** Schematic representation of the experiments. **(B)** Cell viability measured by ATPlite in ES cells after treatment with 5 µM and 10 µM cisplatin at 4 h, 8 h and 24 h of treatment. **(C)** Apoptosis measured by FACS analysis after 8 h and 24 h of treatment with 5 µM cisplatin. **(D)** Cell cycle profile after 8 h and 24 h treatment with PBS or 5 µM cisplatin.
(PDF)

Figure S2 Expanded region between 5.5 and 2.5 ppm of a 1 HNMR spectrum of the apolar extract of HM1 ESC after 8 h of exposure to cisplatin (blue) and to vehicle (red). Arrows indicate characteristic phospholipid signals.
(PDF)

Figure S3 **PCA of the aligned UHPLC-Orbitrap-MS data after ANOVA (p<0.01) and false discovery correction using the Benjamini & Hochberg procedure.** 293 out of 21173 mass peaks survive the ANOVA plus false discovery correction. Green = Control 4 h; Purple = Control 8 h; Red = Cisplatin 4 h; Yellow = Cisplatin 8 h.
(PDF)

Figure S4 **Metscape “gene-compound metabolic network”.** Highlighted in blue and red are compounds and genes showing a significant regulation after 4 h cisplatin treatment. Metabolic enzymes were retrieved from this network (Fig. 2A, Suppl. Table 2). Figure is high resolution – zoom in to view details.
(PDF)

Figure S5 **(A) Regulation of (de)methylases.** Heatmap showing regulation of methyltransferases and demethylases after cisplatin treatment **(B) ROS formation is caused by hydrogen peroxide but not cisplatin treatment.** Bar graph shows normalized fluorescence indicating intracellular ROS levels measured using 40 µM DCF-DA probe. Cells were preincubated with DCF-DA for 1 h and exposed to 5 µM cisplatin or 250 µM H_2_O_2_ in the presence or absence of 10 mM of the ROS scavenger NAC for the indicated times. Bars represent average and SEM of at least 3 independent experiments.
(PDF)

Table S1 **Identified metabolites.** Identification of masses found to be significantly different (p<0.01) between control and cisplatin-treated samples.
(XLS)

Table S2 **Significantly regulated metabolic enzymes.** List of metabolic enzymes identified by Metscape and Ingenuity pathway analysis from 2269 genes that are differentially regulated by cisplatin.
(XLS)

Material S1 Orbitrap mass spectrometer settings.
(PDF)
