# Supplementary figures and images for: Identification of Cisplatin-Regulated Metabolic Pathways in Pluripotent Stem Cells
Source: PLoS One. 2013 Oct 16;8(10):e76476. doi: 10.1371/journal.pone.0076476 (PMC3797786; doi:10.1371/journal.pone.0076476)

A

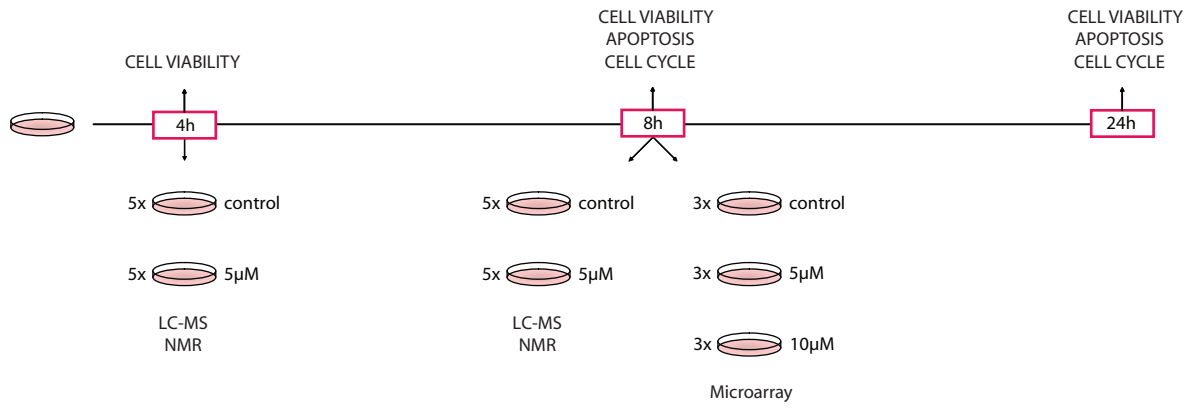

B

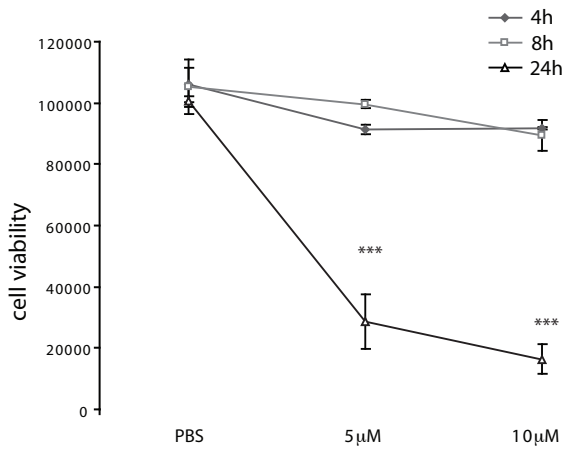

C

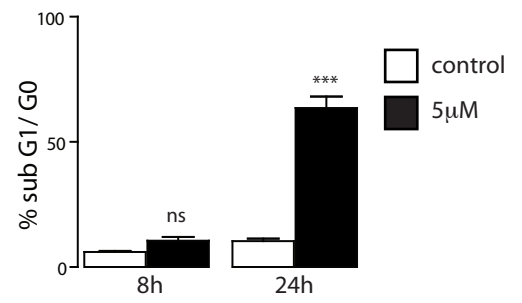

D

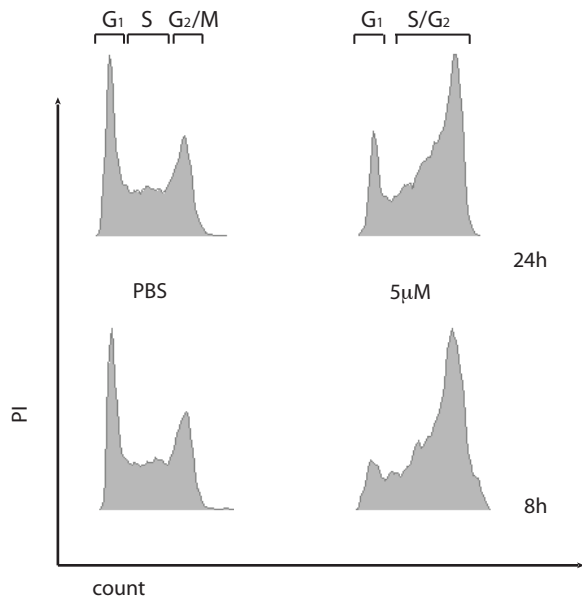

Supplement: Figure S1 — Cisplatin does not lead to cell death at 4 h and 8 h of treatment, but causes cell cycle arrest. (A) Schematic representation of the experiments. (B) Cell viability measured by ATPlite in ES cells after treatment with 5 µM and 10 µM cisplatin at 4 h, 8 h and 24 h of treatment. (C) Apoptosis measured by FACS analysis after 8 h and 24 h of treatment with 5 µM cisplatin. (D) Cell cycle profile after 8 h and 24 h treatment with PBS or 5 µM cisplatin. (PDF) [file pone.0076476.s001.pdf]

Figure S2

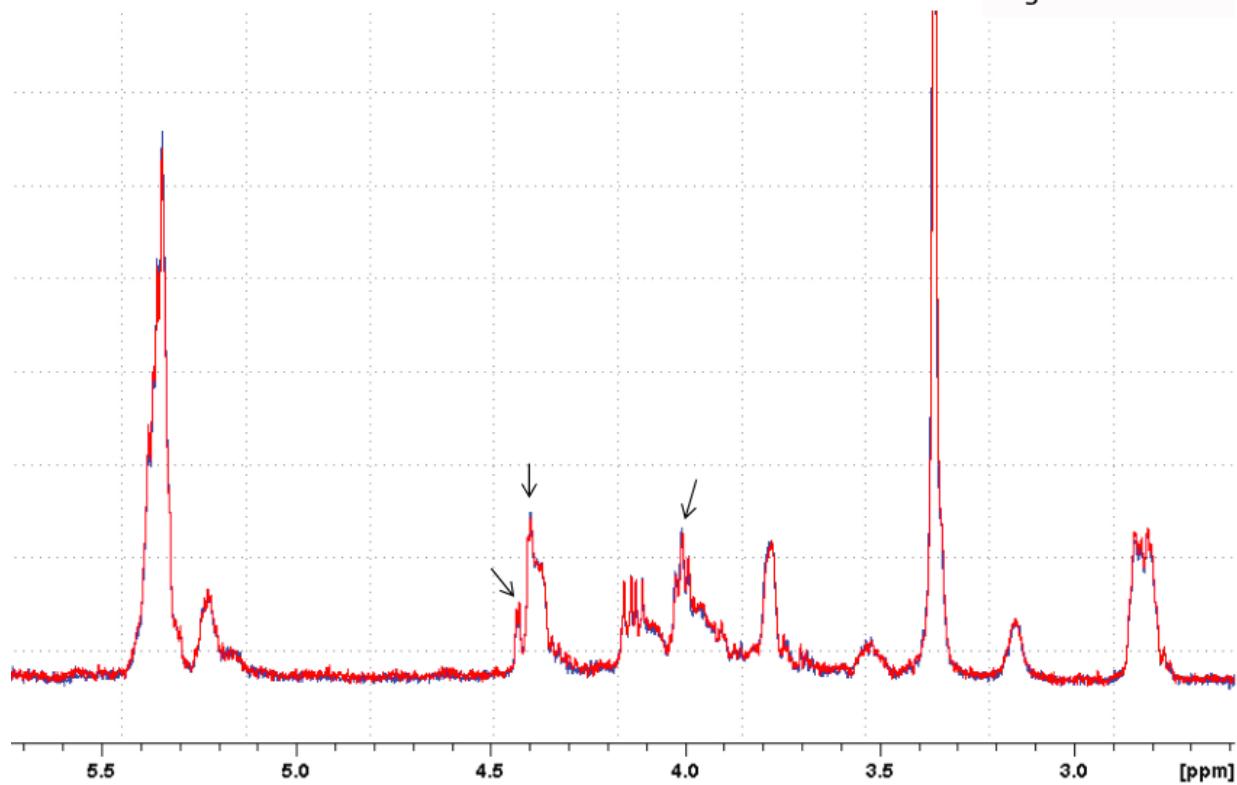

Supplement: Figure S2 — Expanded region between 5.5 and 2.5 ppm of a 1 HNMR spectrum of the apolar extract of HM1 ESC after 8 h of exposure to cisplatin (blue) and to vehicle (red). Arrows indicate characteristic phospholipid signals. (PDF) [file pone.0076476.s002.pdf]

FigureS3

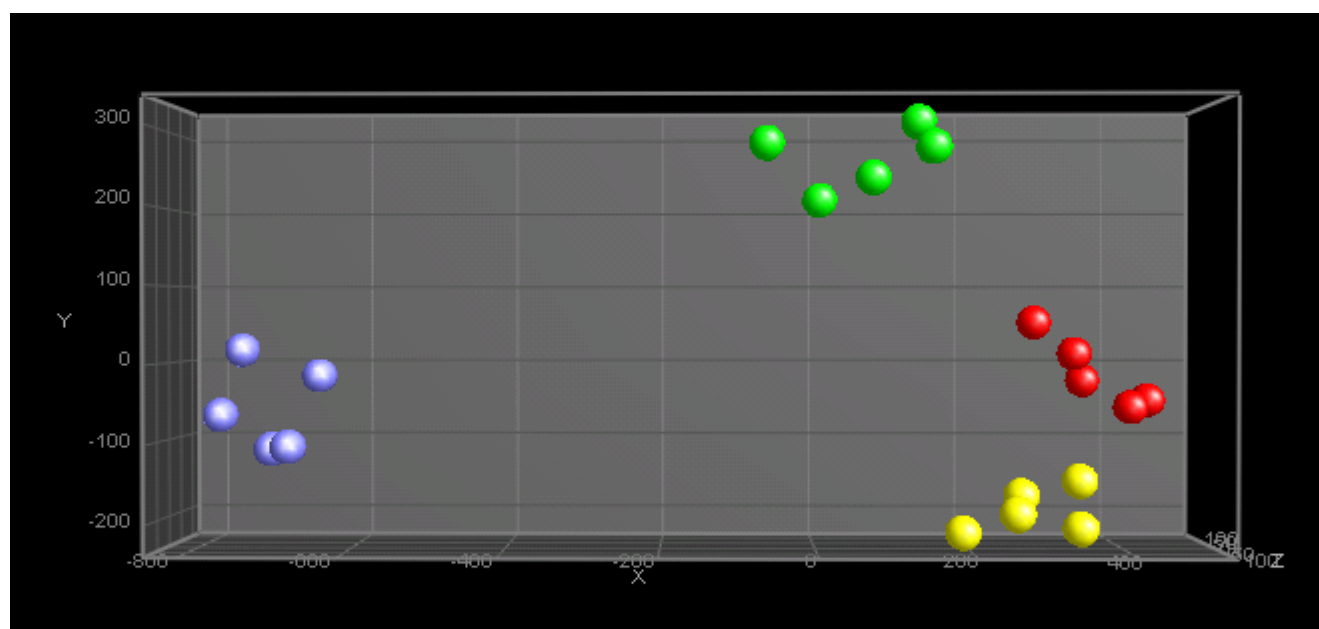

Supplement: Figure S3 — PCA of the aligned UHPLC-Orbitrap-MS data after ANOVA (p<0.01) and false discovery correction using the Benjamini & Hochberg procedure. 293 out of 21173 mass peaks survive the ANOVA plus false discovery correction. Green = Control 4 h; Purple = Control 8 h; Red = Cisplatin 4 h; Yellow = Cisplatin 8 h. (PDF) [file pone.0076476.s003.pdf]

FigureS4

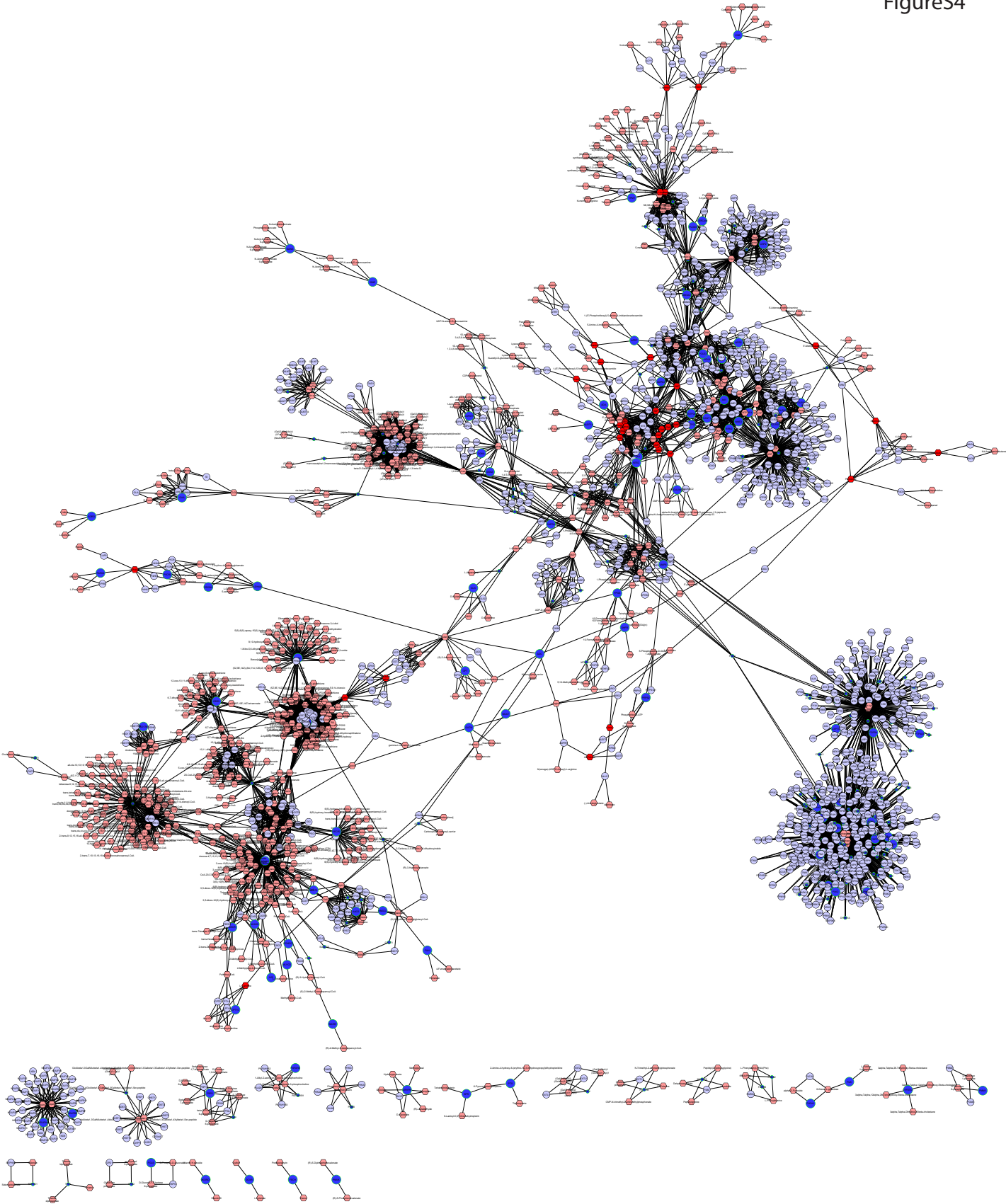

Supplement: Figure S4 — Metscape “gene-compound metabolic network”. Highlighted in blue and red are compounds and genes showing a significant regulation after 4 h cisplatin treatment. Metabolic enzymes were retrieved from this network (Fig. 2A, Suppl. Table 2). Figure is high resolution – zoom in to view details. (PDF) [file pone.0076476.s004.pdf]

A

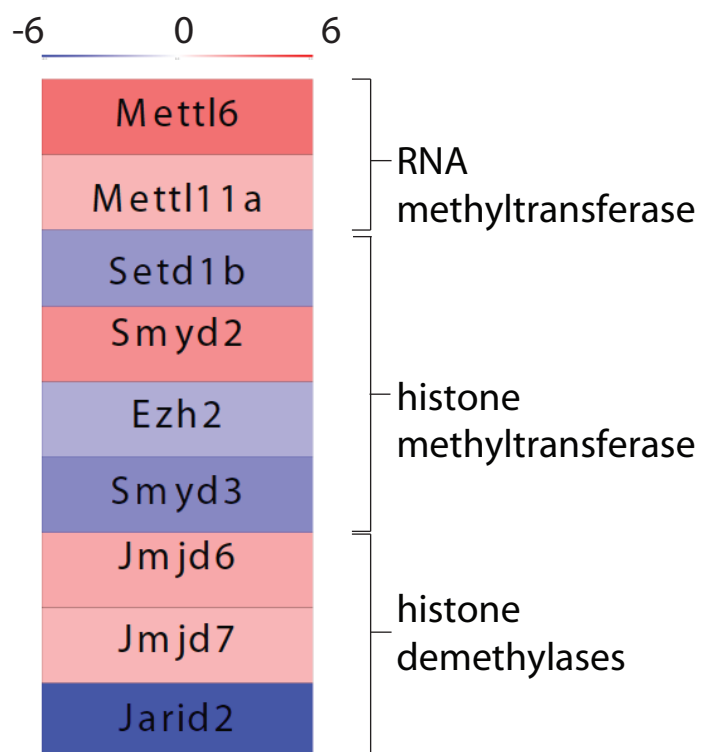

B

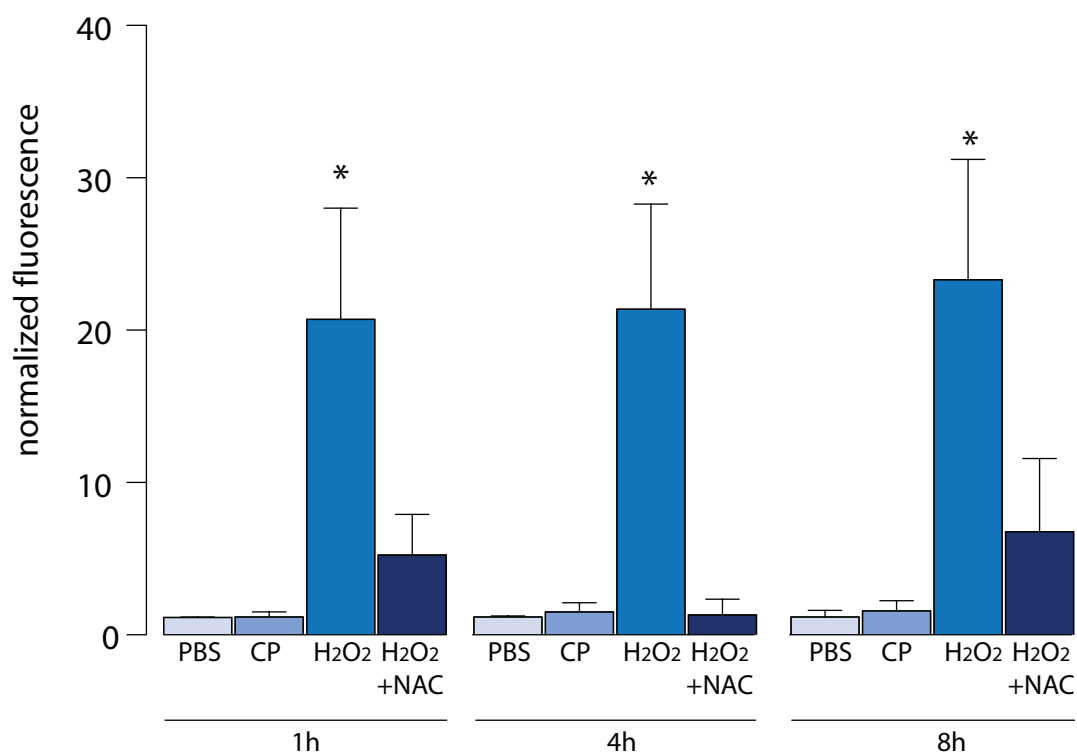

Supplement: Figure S5 — (A) Regulation of (de)methylases. Heatmap showing regulation of methyltransferases and demethylases after cisplatin treatment (B) ROS formation is caused by hydrogen peroxide but not cisplatin treatment. Bar graph shows normalized fluorescence indicating intracellular ROS levels measured using 40 µM DCF-DA probe. Cells were preincubated with DCF-DA for 1 h and exposed to 5 µM cisplatin or 250 µM H2O2 in the presence or absence of 10 mM of the ROS scavenger NAC for the indicated times. Bars represent average and SEM of at least 3 independent experiments. (PDF) [file pone.0076476.s005.pdf]
