# Supplementary material for: Identification of Cisplatin-Regulated Metabolic Pathways in Pluripotent Stem Cells
Source: PLoS One. 2013 Oct 16;8(10):e76476. doi: 10.1371/journal.pone.0076476 (PMC3797786; doi:10.1371/journal.pone.0076476)
Supplement: Material S1 — Orbitrap mass spectrometer settings. (PDF) [file pone.0076476.s008.pdf]

Suppl. Material 1

Orbitrap Mass Spectrometer settings

Heated electrospray interface (HESI): operating in positive mode (ESI^+).

Data acquisition: between m/z 100 and m/z 1000

Resolving power: 50.000 (FWHM)

Scan time: 0.5 s

Spray voltage: 2800 V

Capillary voltage: 47.5 V

Capillary temperature: 250 ˚C

Sheath gas flow: 19 arbitrary units

Auxiliary gas flow: 7 arbitrary units

Instrument calibration: externally, prior to sequence by infusion of calibration solution (m/z 138 to m/z 1822) containing caffeine, MFRA (Met-Arg-Phe-Ala), ultramark 1621, acetic acid in acetonitrile/methanol/water (2:1:1, v/v) (Sigma-Aldrich).
